# AAclust: *k*-optimized clustering for selecting redundancy-reduced sets of amino acid scales

**DOI:** 10.1093/bioadv/vbae165

**Published:** 2024-10-30

**Authors:** Stephan Breimann, Dmitrij Frishman

**Affiliations:** Department of Bioinformatics, School of Life Sciences, Technical University of Munich (TUM), Freising, 85354, Germany; Division of Metabolic Biochemistry, Biomedical Center (BMC), LMU Munich, Munich, 81377, Germany; Biochemistry of γ-Secretase, German Center for Neurodegenerative Diseases (DZNE), Munich, 81377, Germany; Department of Bioinformatics, School of Life Sciences, Technical University of Munich (TUM), Freising, 85354, Germany

## Abstract

**Summary:**

Amino acid scales are crucial for sequence-based protein prediction tasks, yet no gold standard scale set or simple scale selection methods exist. We developed AAclust, a wrapper for clustering models that require a pre-defined number of clusters *k*, such as *k*-means. AAclust obtains redundancy-reduced scale sets by clustering and selecting one representative scale per cluster, where *k* can either be optimized by AAclust or defined by the user. The utility of AAclust scale selections was assessed by applying machine learning models to 24 protein benchmark datasets. We found that top-performing scale sets were different for each benchmark dataset and significantly outperformed scale sets used in previous studies. Noteworthy is the strong dependence of the model performance on the scale set size. AAclust enables a systematic optimization of scale-based feature engineering in machine learning applications.

**Availability and implementation:**

The AAclust algorithm is part of AAanalysis, a Python-based framework for interpretable sequence-based protein prediction, which is documented and accessible at https://aaanalysis.readthedocs.io/en/latest and https://github.com/breimanntools/aaanalysis.

## 1 Introduction

Starting with the influential works of Sneath (1966), Bigelow (1967), Zimmerman *et al.* (1968) in the 1960s, amino acids have been described by numerical indices or scales reflecting their physicochemical properties, such as volume, polarity, or charge. The AAindex database (Kawashima *et al.* 2008) currently contains 566 experimentally measured or computationally derived indices published in 149 studies over six decades. Since the first version of AAindex in 1988 (Nakai *et al.* 1988), this database has been a valuable source for general protein bioinformatic tools based on biophysical properties, such as the ExPASy server (Gasteiger *et al.* 2005), and for scale-based feature engineering in machine learning (Chen *et al.* 2018, Liu *et al.* 2019, Chen *et al.* 2021). However, the AAindex database is highly redundant—e.g., over 120 scales are dedicated to polarity and α-helix propensity. While subsets of AAindex (Gasteiger *et al.* 2005, Chen *et al.* 2018, Liu *et al.* 2019, Chen *et al.* 2021) are commonly used in sequence-based machine learning applications, typically selected based on heuristic criteria, a universally accepted “gold-standard” scale set has so far been lacking.

Redundancy increases the data dimensionality, leading potentially to a bias toward repetitive information and overfitting in machine learning applications (Pudjihartono *et al.* 2022). Reducing such redundancies can improve the efficiency and performance of algorithms, while also enhancing their general interpretability (Ding and Peng 2005). Redundancy reduction is a common step in a variety of bioinformatics applications, such as summarizing gene ontology (Ashburner *et al.* 2000) term lists (e.g., via REVIGO (Supek *et al.* 2011)), or creating redundancy-reduced protein sequence sets (e.g., via CD-HIT (Fu *et al.* 2012)). These methods are designed to cluster data based on similarity measures, such as semantic or sequence similarity, and then select a single representative per cluster. In this vein, we introduce AAclust, a clustering framework leveraging Pearson correlation as a similarity measure to select redundancy-reduced amino acid scale sets. Using machine learning models, we assessed the performance of AAclust scale selections against “gold standard,” randomly selected, and principal component (PC)-based scales.

## 2 Material and methods

### 2.1 Dataset collation

#### 2.1.1 Amino acid scales

We assembled a set of 586 amino acid scales (SCALES, Supplementary Table S1) by first obtaining 553 scales from AAindex that do not contain missing values. We included 21 further scales regarding accessible surface area from Lins *et al.* (2003) and 12 hydrophobicity scales from Koehler *et al.* (2009) because of their relevance for protein folding (Savojardo *et al.* 2021) and backbone dynamics (Quint *et al.* 2010). Each scale was min–max normalized to the range of [0, 1].

#### 2.1.2 Scale sets for benchmarking

Scale sets selected by AAclust were compared against three groups of baseline scale sets:

- *standard*: Three “gold standard” sets, two from previous studies comprising 7 (Meiler *et al.* 2001, Tang *et al.* 2021) and 12 (Pazos 2021) scales (Supplementary Table S1), and all 586 scales from SCALES.
- *pc-based*: All scales from SCALES transformed using PC analysis into 20 PCs, each serving as a single scale, named P1–P20.
- *random*: Subsets of varying size assembled by randomly sampling scales from SCALES.

#### 2.1.3 Datasets of protein sequences

We collated 12 protein sequence datasets (Supplementary Tables S1 and S2) from previous studies targeting distinct binary classification tasks: six datasets were used to predict entire protein sequence properties, while the other six served to predict residue properties in specific sequence positions. These groups are referred to as “sequence prediction” and “residue prediction” dataset, respectively, with individual dataset names based on the prediction task (“SEQ_*dataset_name*” or “AA_*dataset_name*”). For residue predictions, we used three amino acid window sizes (*n* = 5, 9, 13), resulting in a total of 24 benchmark datasets. Each benchmark dataset was balanced by randomly sampling 400 data points without replacement for each class at either the protein or residue level, yielding a total of 800 samples per dataset. Classes in our binary prediction tasks are defined as positive (labelled 1) and negative (labelled 0), such as soluble versus insoluble proteins. See Supplementary Table S2 for details on the class labelling.

### 2.2 AAclust: *k*-optimized clustering

AAclust is a clustering wrapper (Talavera 2005, Solorio-Fernández *et al.* 2016) framework extending clustering models that require a pre-defined number of clusters *k*, such as *k*-means (MacQueen 1967), thereby eliminating the need to specify *k* in advance. It automatically partitions scale sets into *k* clusters by maximizing the within-cluster Pearson correlation to surpass a user-defined minimum threshold *min_th*. Two alternative quality measures are employed: the minimum pairwise Pearson correlation among all cluster members (“*min_cor_all_*”) or the minimum Pearson correlation between the cluster center and all cluster members (“*min_cor_center_*”). The minimum correlation across all clusters can be maximized using either *min_cor_all_* or *min_cor_center_*.

**Figure 1.**
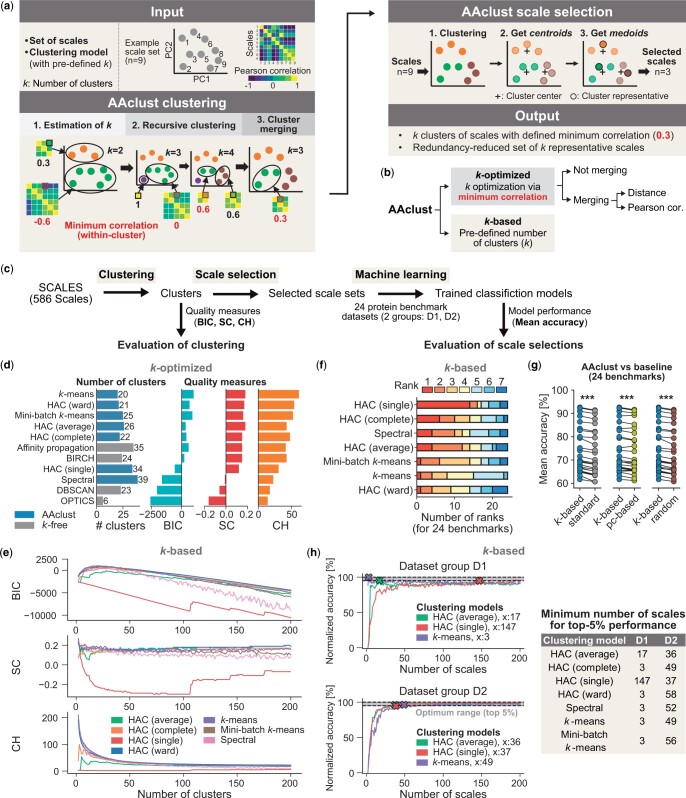
AAclust algorithm, workflow, and evaluation. (**A–C**) Overview of the AAclust algorithm, settings, and evaluation workflow. (**D**, **E**) Evaluation of AAclust clustering using Bayesian information criterion (BIC), silhouette coefficient (SC), and the Calinski Harabasz score (CH). (**D**) Comparison of the best *k*-optimized AAclust approaches against *k*-free clustering models. (**E**) Relation between the number of clusters and the quality measures for *k*-based AAclust approaches. (**F**, **G**) Evaluation of AAclust scale selection. (**F**) The number of ranks of best-performing clustering models for *k*-based AAclust approaches. (**G**) Comparison of best-performing (for each benchmark dataset) *k*-based AAclust approaches against baseline scale sets: “standard” (gray), “pc-based” (yellow), and “random” (brown). Differences were tested by paired Wilcoxon signed-rank test, Benjamini-Hochberg correction (*P < .05, **P < .01, ***P < .001). (**H**) Relation between the number of scales and the aggregated prediction performance for *k*-based AAclust approaches. “x: *n*” indicates the minimum number of scales *n* for a top-5% performance.

Optimizing *k* in a three-step procedure (Fig. 1a), AAclust first estimates the lower bound of *k*, then refines it through recursive clustering (using *min_cor_all_* or *min_cor_center_*), and optionally merges smaller clusters into larger ones based on Pearson correlation or Euclidean distance. AAclust is controlled by three parameters:

- *min_th*: Sets the Pearson correlation threshold (between 0 and 1, default *min_th* = 0.3) to define the minimum correlation for all clusters.
- *Center*: Determines whether *min_th* applies to the cluster center (true) or all cluster members (false), using either *min_cor_center_* or *min_cor_all_*, respectively.
- *Merge*: Enables (true) or disables (false) the optional merging step.

To obtain redundancy-reduced scale sets, AAclust selects one representative scale per cluster, closest to its center. Alternatively, users can set *k* to a pre-defined number of scales. Both methods are referred to as “*k*-optimized” and “*k*-based” approaches (Fig. 1b), respectively.

### 2.3 Quality measures of clustering

To evaluate cluster quality (Fig. 1c), we clustered all scales from SCALES by seven clustering models used with the *k*-optimized AAclust approach and three clustering models that do not require a pre-specified *k*, referred to as “*k*-free” clustering models (Supplementary Table S2). Three commonly used clustering quality measures (Wiwie *et al.* 2015, Ronan *et al.* 2016, Ahmad and Khan 2019) were employed: silhouette coefficient (SC) (Rousseeuw 1987,  Kaufman and Rousseeuw 2009), Calinski Harabasz score (CH) (Calinski and Harabasz 1974), and Bayesian information criterion (BIC) (Schwarz 1978) (Supplementary Table S3).

### 2.4 Evaluation procedure for scale selections

To assess AAclust scale selections (Fig. 1c), we compared them to “standard,” “pc-based,” and “random” scale sets. AAclust was employed with seven clustering models, testing different *k*-optimized AAclust settings and assessing various scale set ranges for *k*-based approaches.

Each scale set served as a feature set for 24 benchmark datasets using three machine learning models with default settings: random forest, support vector machine, and logistic regression. Model performance was measured by accuracy (ACC) using five-fold cross validation. To minimize model-dependent bias, we averaged accuracy across all folds and models (“mean accuracy”), using it as the quality measure for each scale set.

## 3 Results

### 3.1 Evaluation of clustering

We comprehensively evaluated the quality of clusters derived from the entire SCALES dataset by AAclust approaches and *k*-free clustering models (Supplementary Fig. S1) using the BIC, SC, and CH quality measures. We first optimized *k*-optimized AAclust settings for seven models, such as hierarchical agglomerative clustering (HAC). The best performance was achieved by *k*-means when using merging, *min_cor_center_*, and *min_th* = 0. Five of the seven *k*-optimized AAclust approaches outperformed *k*-free models (Fig. 1d), with the highest BIC scores around 25 clusters, declining linearly thereafter (Fig. 1e). Comparing all *k*-optimized against *k*-based approaches (with equal number of clusters) showed a significant (*P* < .001) impact of merging on the clustering quality, improving BIC but worsening CH, while *k*-based approaches generally enhanced SC and CH (Supplementary Fig. S1).

The difference between the *k*-optimized and *k*-based AAclust settings are exemplified for the HAC clustering models and 100 randomly selected scales (Supplementary Fig. S1b). Without merging, seven clusters of varied size with an interquartile range (IQR) of 1–25 scales are obtained. Applying merging in a *k*-optimized setting yields four evenly sized clusters with an IQR of 22–28 scales, while using the *k*-based approach with *k* = 4 forms two large and two small clusters only comprising 1 and 2 scales. Since AAclust selects for each cluster one representative scale, the merging step has a significant impact on the subsequent scale selection.

### 3.2 Evaluation of scale selection

We compiled 24 benchmark datasets (Supplementary Fig. S2), including 6 for “sequence prediction” and 18 for “residue prediction,” to assess AAclust scale selections (Supplementary Fig. S3). These selections were used as features for machine learning models, with their average accuracy (“mean accuracy”) on the benchmark datasets indicating the quality of the scale set.

#### 3.2.1 Evaluation of AAclust scale selection

We evaluated *k*-based AAclust scale selections by obtaining scale sets of size ranging between 2 and 585 scales for seven clustering models (Supplementary Fig. S3). The top-performing approaches were ranked for each dataset separately, showing similar mean accuracy values. The best-ranked models had the fewest scales (median: 103, IQR: 50–167), and the model rank positively correlated with the number of scales (Spearman’s correlation = 0.16, *P* < .05). Some clustering models, such as HAC (single), performed weakly in clustering (Fig. 1d and e) but well in prediction (Fig. 1f), while others exhibited the opposite trend, such as *k*-means.

AAclust-based strategies (*k*-optimized and *k*-based) significantly improved mean accuracy over the three baseline scale sets (Fig. 1g, Supplementary Fig. S4). Notably, *k*-based approaches had a slightly better performance than *k*-optimized approaches, albeit with higher variability (Supplementary Table S4). For most datasets and scale set ranges, *k*-based AAclust approaches outperformed randomly sampled sets (Supplementary Fig. S3). However, pc-based sets were superior for ranges with *n* ≤ 20 scales.

#### 3.2.2 Correlation of clustering quality and prediction performance

For the *k*-based AAclust approaches, we examined correlations between clustering quality, the number of scales, and the scale set quality (as quantified by machine learning model performance). The prediction performance was averaged across seven clustering models for each scale set size and benchmark dataset (MEAN_ACC_*dataset*). We then hierarchically clustered these 24 benchmark datasets into two groups (D1 and D2, Supplementary Fig. S5).

We aggregated the model performance for D1 and D2 (“ACC|D1” and “ACC|D2”) and explored Pearson correlations across four scale set ranges. Correlations between the model performance and the number of scales were mainly positive, particularly for the 2–29 range and D2. For larger scale set ranges, these correlations diverged, varying by clustering model and dataset. After min–max normalization, most clustering models achieved a normalized accuracy ≥ 95% for D1 with few scales (e.g., 3 for *k*-means), while more than 35 were required for D2 (Fig. 1h). Remarkably, pc-based sets achieved optimal results with just 5 scales (i.e., the first PCs) for D1, but required all 20 for D2 (Supplementary Fig. S6).

#### 3.2.3 Effect of AAclust settings on prediction performance

We evaluated the impact of *k*-optimized AAclust settings on prediction performance for dataset groups D1 (ACC|D1) and D2 (ACC|D2). Generally, prediction performance was lower for D1 than for D2, and the best results for D2 were obtained with *min_th* between 0 and 0.6 without merging, where HAC (average) for D1 and *k*-means for D2 performed best (Supplementary Fig. S7).

Analyzing cluster merging showed that *k*-based approaches performed significantly (*P* < .001) lower for D1, while for D2, *k*-based and *k*-optimized (without merging) approaches were significantly better (*P* < .001) than those using merging. Disabling the “Center” parameter significantly (*P* < .01–.001) improved D2 performance. Assessing merging impact on min–max normalized accuracy values showed that smaller scale sets are preferred for D1 and larger for D2, consistent with prior results (Fig. 1h). Overall, our results emphasize that the optimal scale selection depends on the clustering model and the protein dataset.

### 3.3 Use case of optimizing scale selections by AAclust

To illustrate the application of AAclust, we present a short case study for a viral capsid dataset (“SEQ_CAPSID”), part of our sequence prediction benchmark datasets (Supplementary Tables S1 and S2). This dataset was utilized for the binary classification of viral capsid (and non-capsid) proteins, which form a shell enclosing viral genetic material, crucial for sequence annotation in metagenomic projects (Galiez *et al.* 2016).

Each protein was represented by a vector of length *k*, where *k* is the number of used scales (clusters), and each value corresponds to the average value of a selected scale over its complete sequence. We tested different sizes of *k*, ranging from 2 to 29 scales, and compared the prediction performance of classical machine learning models, such random forest or support vector machine, against the number of scales and three clustering quality measures: BIC, SC, and CH (Supplementary Fig. S5d).

The results demonstrated cluster model-specific differences. For example, approaches based on HAC (average) showed slightly better performance compared to *k*-means. Additionally, the correlation between accuracy and CH was positive for HAC (average) and negative for *k*-means within the 2–29 scale set range. This case study underscores the practical utility of AAclust in comparing optimized feature sets for scale-based protein prediction tasks.

### 3.4 AAclustTop60

We compiled the 60 best scale sets from all AAclust approaches into “AAclustTop60” (Supplementary Fig. S8 and Supplementary Table S3). This collection comprises 48 top-ranked sets for 24 benchmark datasets (Supplementary Table S4) and 12 top-ranked sets for dataset groups D1 and D2. We ranked these sets by average prediction performance and clustering quality, showing an anti-correlation (Pearson’s *r* = −0.77, *P* < .01), whereby the number of scales correlated negatively with the former and positively with the latter. On average, sets in AAclustTop60 contained 125 ± 121 scales (median: 98, IQR: 48–154) obtained using various AAclust settings. The variation in AAclustTop60 underlines that the optimal scale set depends on the protein prediction tasks.

## 4 Implementation

AAclust is integrated in AAanalysis, a Python framework for interpretable sequence-based protein prediction. Besides AAclust, AAanalysis also provides the complete scale sets (SCALES), the 12 protein datasets, and the AAclustTop60.

For systematic optimization of sequence-based feature engineering using AAclust, we recommend the following steps:

1. Test all sets of AAclustTop60 as distinct features to establish baseline models and identify the best *k*-optimized AAclust settings, clustering models, and scale set ranges.
2. For the best clustering models, test (a) *k*-optimized AAclust approaches encompassing the best settings, and (b) *k*-based AAclust approaches within the optimal scale set range.
3. If the optimal range is within 2–20 scales, test pc-based scale sets.

Alternatively, AAclustTop60 scale sets could serve as initial population for genetic algorithms to optimize feature engineering (Telikani *et al.* 2022). Clustering models compatible with AAclust require a pre-defined number of clusters and should be implemented in scikit-learn or work accordingly.

## 5 Conclusion

We introduced AAclust, a clustering wrapper framework for selecting redundancy-reduced amino acid scale sets. Using Pearson correlation, AAclust optimizes the number of clusters and selects one scale per cluster. Our benchmarking experiments show that (a) no single “gold standard” scale set exists, (b) the scale set size is a crucial and dataset-dependent optimization factor, and (c) AAclust scale selections significantly improve the performance of machine learning methods. Additionally, we collated the 60 best-performing scale sets (AAclustTop60) and provided a three-step application guide.

Although scale-based machine learning approaches have limitations (Raimondi *et al.* 2019), particularly in performance compared to deep learning-based protein embeddings (i.e., scale-like residue representations generated using protein large language models such as ProtT5 (Elnaggar *et al.* 2022)), their advantage lies in their interpretability, which remains challenging for deep learning models (Greener *et al.* 2022). Overall, AAclust tailors scale sets to specific protein prediction tasks, enabling systematic and interpretable sequence-based feature engineering.

## Supplementary Material

vbae165_Supplementary_Data

vbae165_Supplementary_Material

## Data Availability

The data underlying this article are available through the AAanalysis API at https://aaanalysis.readthedocs.io/en/latest/generated/aaanalysis.load_dataset.html.
