# Supplementary material for: AAclust: k-optimized clustering for selecting redundancy-reduced sets of amino acid scales
Source: Bioinform Adv. 2024 Oct 30;4(1):vbae165. doi: 10.1093/bioadv/vbae165 (PMC11562964; doi:10.1093/bioadv/vbae165)
Supplement: vbae165_Supplementary_Data [file vbae165_supplementary_data.zip › FigS2.pdf]

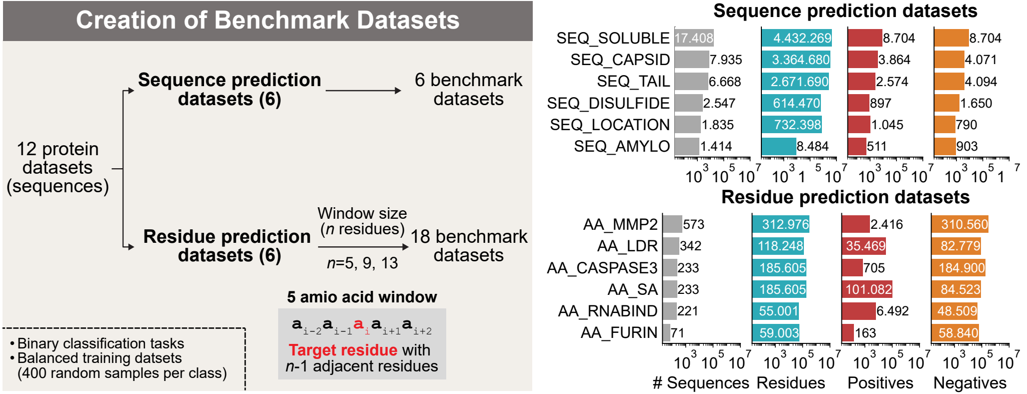

Creation of Benchmark Datasets
12 protein datasets (sequences)
Sequence prediction datasets (6)
6 benchmark datasets
Residue prediction datasets (6)
Window size (n residues)
n=5, 9, 13
18 benchmark datasets
5 amio acid window
a i−2 a i−1 a i a i+1 a i+2
Target residue with n-1 adjacent residues
• Binary classification tasks
• Balanced training datsets (400 random samples per class)
Sequence prediction datasets
SEQ_SOLUBLE 17.408 4.432.269 8.704 8.704
SEQ_CAPSID 7.935 3.364.680 3.864 4.071
SEQ_TAIL 6.668 2.671.690 2.574 4.094
SEQ_DISULFIDE 2.547 614.470 897 1.650
SEQ_LOCATION 1.835 732.398 1.045 790
SEQ_AMYLO 1.414 8.484 511 903
Residue prediction datasets
AA_MMP2 573 312.976 2.416 310.560
AA_LDR 342 118.248 35.469 82.779
AA_CASPASE3 233 185.605 705 184.900
AA_SA 233 185.605 101.082 84.523
AA_RNABIND 221 55.001 6.492 48.509
AA_FURIN 71 59.003 163 58.840
# Sequences
Residues
Positives
Negatives
