# Supplementary figures and images for: AAclust: k-optimized clustering for selecting redundancy-reduced sets of amino acid scales
Source: Bioinform Adv. 2024 Oct 30;4(1):vbae165. doi: 10.1093/bioadv/vbae165 (PMC11562964; doi:10.1093/bioadv/vbae165)

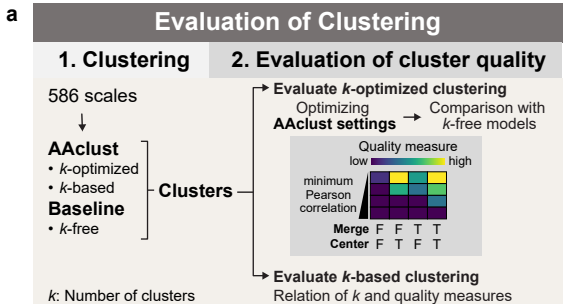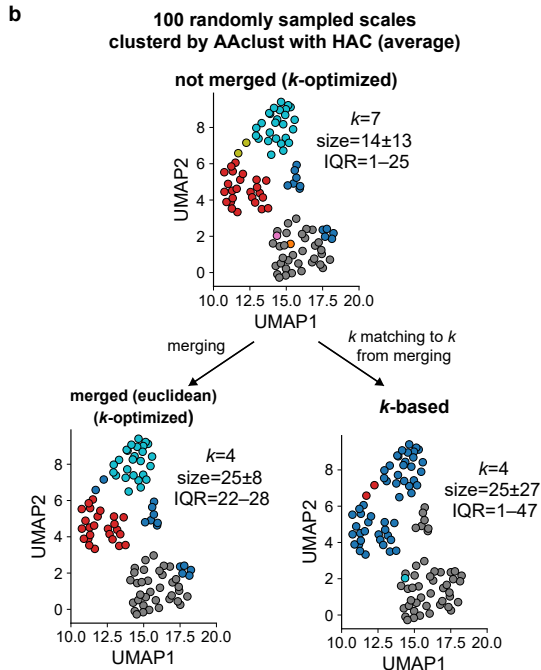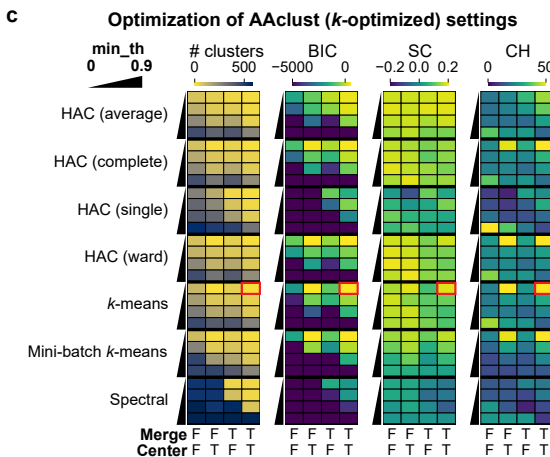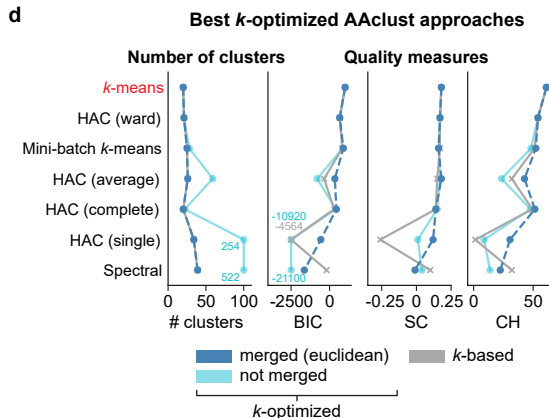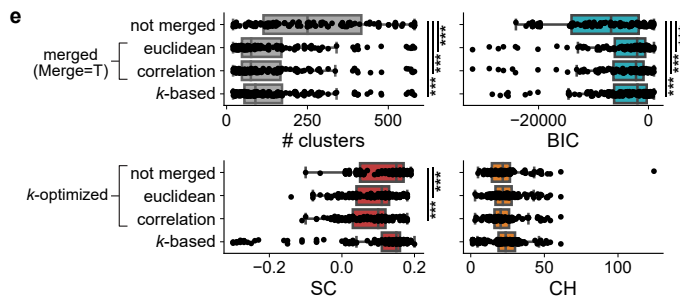

Supplement: vbae165_Supplementary_Data [file vbae165_supplementary_data.zip › FigS1.pdf]

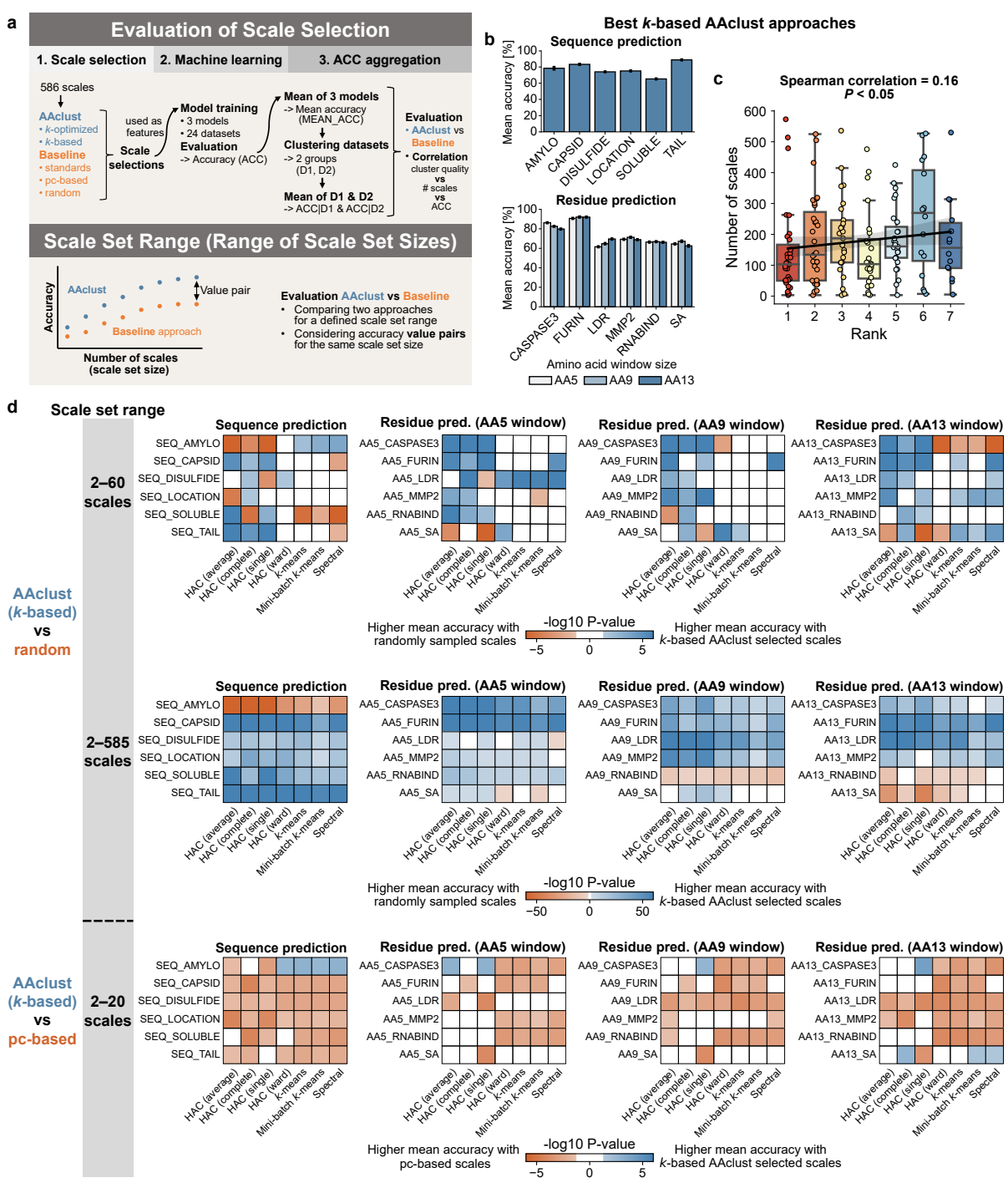

Supplement: vbae165_Supplementary_Data [file vbae165_supplementary_data.zip › FigS3.pdf]

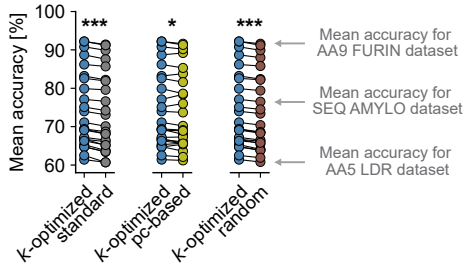

Supplement: vbae165_Supplementary_Data [file vbae165_supplementary_data.zip › FigS4.pdf]

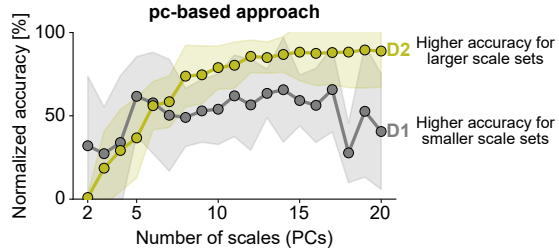

Supplement: vbae165_Supplementary_Data [file vbae165_supplementary_data.zip › FigS6.pdf]

# a Optimization of AAclost (*k*-optimized) settings

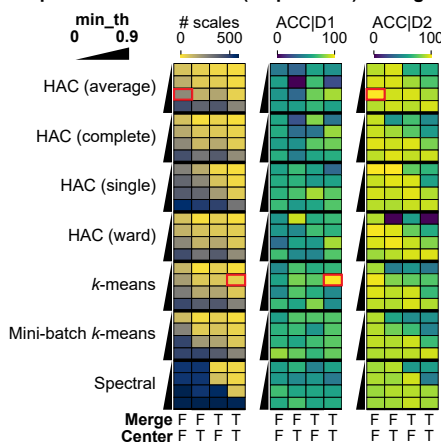

b

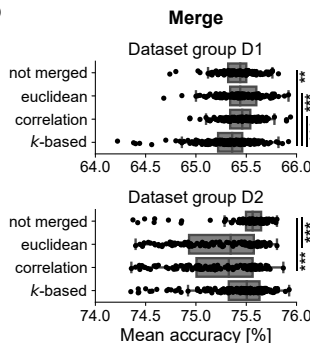

c

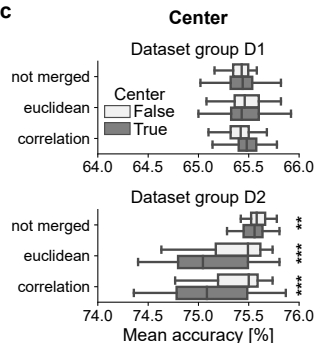

d

## All *k*-optimized AAclost approaches

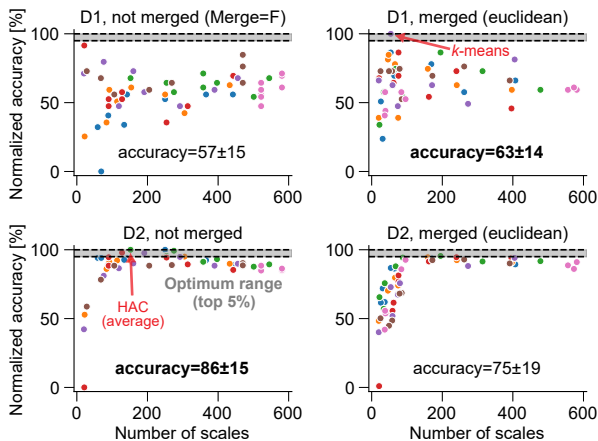

Supplement: vbae165_Supplementary_Data [file vbae165_supplementary_data.zip › FigS7.pdf]

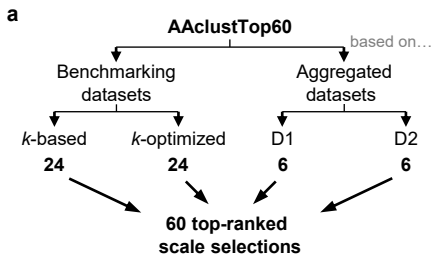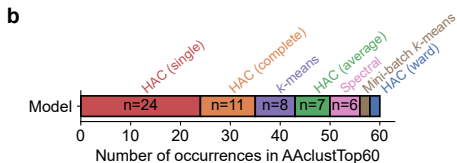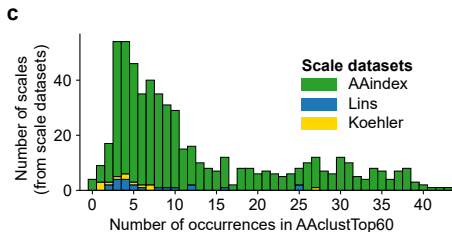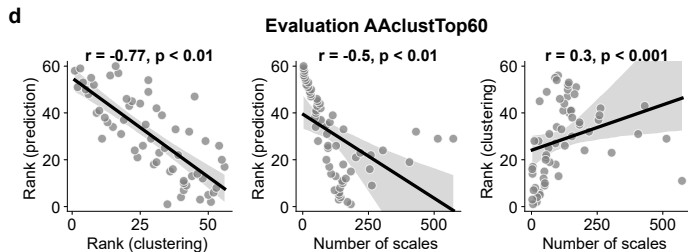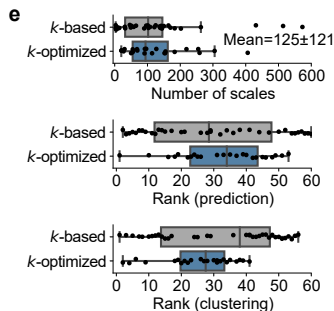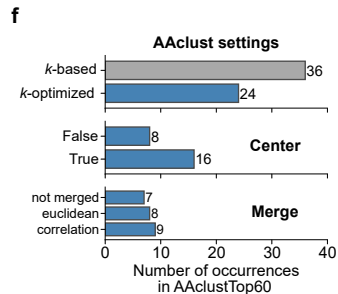

Supplement: vbae165_Supplementary_Data [file vbae165_supplementary_data.zip › FigS8.pdf]
